# Inducible HIV-1 Reservoir Reduction Assay (HIVRRA), a Fast and Sensitive Assay to Test Cytotoxicity and Potency of Cure Strategies to Reduce the Replication-Competent HIV-1 Reservoir in Ex Vivo PBMCs

**DOI:** 10.21769/BioProtoc.5384

**Published:** 2025-07-20

**Authors:** Jade Jansen, Teunis B. H. Geijtenbeek, Neeltje A. Kootstra

**Affiliations:** 1 Department of Experimental Immunology, Amsterdam UMC, University of Amsterdam, Amsterdam, The Netherlands; 2 Amsterdam Institute for Immunology and Infectious Diseases, Amsterdam, The Netherlands

**Keywords:** Inducible HIV-1 reservoir reduction assay (HIVRRA), HIV-1, HIV-1 reservoir, TZM-BL cells, Latency, Cytotoxicity, HIV-1 latency reactivation/silencing, PBMCs, People living with HIV-1 (PWH)

## Abstract

The HIV-1 reservoir, consisting of transcriptionally silent integrated HIV-1 proviruses, is a major barrier to a cure, as it persists during effective antiretroviral therapy (ART) and is the source of viral rebound upon treatment interruption. Some of the strategies explored for HIV cure focus on the identification of compounds to either reactivate and eliminate the HIV reservoir (“shock and kill”) or to prevent HIV reservoir reactivation and induce deep proviral latency (“block and lock”). Paramount in developing these HIV-1 cure strategies is determining the effect of the compounds on the size of the inducible HIV-1 reservoir in blood from people living with HIV-1 (PWH). Traditionally, viral outgrowth assays have been the primary method to determine the inducible HIV-1 reservoir in CD4+ T cells from PWH. However, these assays are labor-intensive, time-consuming, and often have low sensitivity. We have recently developed the inducible HIV-1 reservoir reduction assay (HIVRRA), a rapid, cost-effective, and sensitive method to measure the impact of compounds on the inducible replication-competent HIV-1 reservoir in total peripheral blood mononuclear cells (PBMCs) from PWH ex vivo. The HIVRRA simultaneously evaluates the effect of test conditions on the size of the inducible replication-competent HIV-1 reservoir as well as the specificity and toxicity of the test strategy. Using total PBMCs instead of purified CD4+ T cells reduces processing time and resource requirements. This makes the HIVRRA a more practical, scalable tool for evaluating potential HIV-1 cure strategies.

Key features

• The HIVRRA builds on the TZM-BL cell-based assay to quantify the HIV-1 reservoir by Sanyal et al.’s [1] method.

• The HIVRRA uses total PBMCs from PWH to determine infectious units per million cells.

• The HIVRRA requires low PBMC input compared to other reservoir analysis methods.

• The HIVRRA determines the toxicity of the compounds on HIV-1-infected and uninfected cells in the same assay.

## Graphical overview


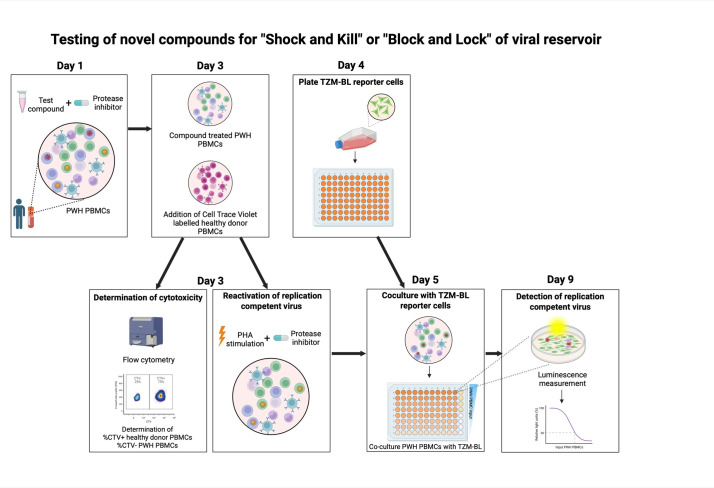


## Background

Antiretroviral therapy (ART) has significantly improved the life of people with HIV-1 (PWH) as it effectively suppresses HIV-1 replication. However, ART does not cure HIV-1 and needs to be taken lifelong, since the virus remains present in the reservoir [2]. This HIV-1 reservoir mainly consists of CD4 memory T cells that harbor an integrated provirus in their genome; because the provirus is transcriptionally latent, these cells are not recognized by the immune system [3,4]. Moreover, when ART is interrupted, HIV-1 replication is initiated from the latent viral reservoir [5,6]. This so-called latent but inducible replication-competent HIV-1 reservoir is the source of viral rebound and remains a significant barrier to achieving a cure [3]. Therefore, cure strategies mainly target the replication-competent viral reservoir. One of these strategies is the “shock and kill” method, which aims at eliminating the viral reservoir using latency-reversing agents (LRAs) to reactivate transcription from the latent provirus; this has the goal to eliminate infected cells either through immune recognition or virus- or compound-induced apoptosis [7]. Another method called “block and lock” specifically aims to permanently silence integrated proviruses, through targeting of viral or host factors essential for HIV-1 transcription [8].

The efficacy of LRAs and compounds used for these strategies has been widely tested in in vitro infection and cell line models [9]. However, it is of great importance that the effectiveness of the compounds used for these strategies is also tested in a more relevant setting using peripheral blood mononuclear cells (PBMCs) from people living with HIV-1 (PWH), measuring the inducible replication-competent viral reservoir. Various methods exist to quantify this reservoir. The gold standard for measuring replication-competent virus remains the quantitative viral outgrowth assay (QVOA). The QVOA gives an estimate of the inducible, replication-competent HIV-1 reservoir by serial diluting CD4+ T cells from PWH and stimulating them with activated healthy PBMCs [stimulation occurs through T-cell receptor cross linking using phytohaemagglutinin (PHA) or an antibody cocktail targeting the T-cell receptor (TCR; CD3) and the costimulatory molecule CD28] [10]. It may take up to 4 weeks before a virus grows to detectable levels, and activated PBMCs need to be added to the culture weekly. This makes the QVOA a time-consuming and labor-intensive method with low sensitivity, underestimating the size of the viral reservoir. Moreover, the QVOA requires a large number of cells from PWH, limiting its feasibility for high-throughput screening applications. There is a need for a fast and sensitive assay to measure the impact of interventions on the inducible HIV-1 reservoir.

Sanyal et al [1] developed the TZM-BL cell-based assay (TZA), an alternative approach to quantify the latent HIV-1 reservoir in blood. In this assay, CD4+ T cells from PWH are isolated, activated, and eventually co-cultured with the TZM-BL reporter cell line. This HeLa-derived cell line can be infected by HIV-1 because it has been modified to express the HIV-1 receptor CD4 and coreceptors CXCR4 and CCR5. Furthermore, it contains the β-galactosidase and firefly luciferase reporter genes under the control of the HIV-1 long terminal repeat (LTR). The LTR contains HIV-1 promoter regions that are activated by the HIV-1 protein TAT, which is produced upon infection with HIV-1 [11]. We modified this assay to utilize PBMCs from PWH as input cells and optimized it to test the effect of different compounds on the inducible viral reservoir, simultaneously assessing cytotoxicity. This inducible HIV-1 reservoir reduction assay (HIVRRA) evaluates the ability of compounds of interest to reduce the replication-competent viral reservoir. To this extent, PBMCs from PWH are incubated with compounds. Subsequently, the treated PBMCs are activated through TCR crosslinking to activate viral transcription of the (remaining) inducible viral reservoir. To prevent viral spread during these steps, a protease inhibitor (like ritonavir, indinavir, or saquinavir) is added during compound incubation and TCR crosslinking. As TCR-stimulation is expected to reactivate a large part of the inducible viral reservoir, our assay is suitable to measure a reduction of the inducible viral reservoir. Compounds that either activate or block viral transcription can be used in our assay and will both result in a reduction of the inducible replication-competent viral reservoir, since transcriptional activators like LRAs will initiate HIV-1-related apoptosis, whereas transcriptional blockers will prevent viral replication. Our HIVRRA method is a rapid, cost-effective, and sensitive method for evaluating the impact of compounds on the inducible replication-competent HIV-1 reservoir.

## Materials and reagents

**Biological materials**

1. TZM-BL (NIH AIDS Reagent program, catalog number: 8129); 2 × 10^6^ cells are required for each condition (compounds or untreated) tested

2. PBMCs from uninfected donors (obtained from blood donors from the Dutch national blood bank in Amsterdam, the Netherlands); 8.2 × 10^5^ cells are required for each condition (compounds or untreated) tested

3. PBMCs from PWH on ART (obtained from Amsterdam Cohort Studies on HIV infection and AIDS); 8.2 × 10^5^ cells are required for each condition (compounds or untreated) tested

**Reagents**

1. Newborn calf serum (NBCS) (ThermoFisher Scientific, catalog number: 16010159)

2. FetalClone II serum (FCS) Cytiva HyClone^TM^ (Fisher Scientific, catalog number: 10326762)

3. Iscove’s modified Dulbecco’s medium (IMDM) (ThermoFisher Scientific, catalog number: 21980065)

4. Phosphate-buffered saline (PBS) (ThermoFisher Scientific, catalog number: 10010023)

5. Hydrochloric acid (HCl), 37% (Sigma-Aldrich, catalog number: 258148)

6. Phytohaemagglutinin (PHA) (ThermoFisher Scientific, catalog number: R30852801)

7. Ciprofloxacin (ThermoFisher Scientific, catalog number: 449620250)

8. Penicillin-Streptomycin (Pen Strep) (ThermoFisher Scientific, catalog number: 15140122), 10,000 U/mL penicillin/10,000 μg/mL streptomycin

9. IL-2 (Miltenyi Biotec, catalog number: 130-097-742)

10. Trypan Blue (Bio-Rad, catalog number: 1450003)

11. Protease inhibitor Saquinavir (MedChemExpress, catalog number: HY-17007), 10 mM in DMSO

12. RQ1 RNase-free DNAse (Promega, catalog number: M6101), 1,000 U/mL

13. CellTrace^TM^ Violet (ThermoFisher Scientific, catalog number: C34557)

14. Fluorofix (BioLegend, catalog number: 422101)

15. Glycerol (Sigma-Aldrich, catalog number: 818709)

16. Triton X-100 (Fisher Scientific, catalog number: 10509020)

17. Dithiothreitol (DTT) (Duchefa Biochemie, catalog number: D1309)

18. D-Luciferin (Duchefa Biochemie, catalog number: L1349)

19. Na_2_H_2_P_2_O_7_ (Sigma-Aldrich, catalog number: 71501)

20. MgCl_2_ (Sigma-Aldrich, catalog number: 814733)

21. ATP (Sigma-Aldrich, catalog number: A2383-1G)

22. NaOH (Sigma-Aldrich, catalog number: 221465)

23. Tris-H_3_PO_4_ (Trizma^®^ phosphate monobasic) (Sigma-Aldrich, catalog number: 93348)

24. DMSO (Sigma-Aldrich, catalog number: 102952)

**Solutions**

1. PHA stock solution (see Recipes)

2. HCl 0.1% (see Recipes)

3. Ciprofloxacin stock solution (see Recipes)

4. IL-2 stock solution (see Recipes)

5. Thaw medium (see Recipes)

6. Cell line medium (see Recipes)

7. Complete medium (see Recipes)

8. Complete medium with protease inhibitor (see Recipes)

9. Complete medium with protease inhibitor and DNAse (see Recipes)

10. PHA medium with protease inhibitor (see Recipes)

11. NaOH (10 M) (see Recipes)

12. Tris-H_3_PO_4_ pH 7.8 (1 M) (see Recipes)

13. Triton X-100 (25%)

14. DTT (1 M) (see Recipes)

15. MgCl_2_ (1 M) (see Recipes)

16. Disodium pyrophosphate (Na_2_H_2_P_2_O_7_) (50 mM) (see Recipes)

17. ATP (50 mM) (see Recipes)

18. Pre-LAR (see Recipes)

19. Pre-LAR + D-Luciferin (see Recipes)

20. LAR substrate (see Recipes)

**Recipes**

**1. PHA stock solution**

Dissolve 2 mg of lyophilized PHA in 10 mL of IMDM at a final concentration of 200 μg/mL. Aliquot and store at -20 °C.

**2. HCl 0.1%**

Add 2.7 mL of HCl 37% to 1,000 mL of MilliQ H_2_O. Store at room temperature (RT).

**Caution**: HCl is a corrosive acid. Handle with care inside a fume hood and use proper personal protection like gloves.

**3. Ciprofloxacin stock solution**

Dissolve 5 mg of Ciprofloxacin in 2.5 mL of 0.1% HCl (Recipe 2) to a final concentration of 2 mg/mL. Aliquot and store at -20 °C.

**4. IL-2 stock solution**

Dissolve 30,000 IU recombinant human IL-2 in 15 mL of IMDM to a final concentration of 2,000 U/mL. Aliquot and store at -20 °C

**5. Thaw medium**

400 mL of IMDM medium and 100 mL of 20% NBCS. Store and keep at 4 °C before use.

**6. Cell line medium**

450 mL of IMDM medium, 50 mL of 10% FCS, 5 mL of 100 U/mL penicillin/100 μg/mL streptomycin, and 1.25 mL of 5 μg/mL ciprofloxacin (see Recipe 3). Store at 4 °C.

**7. Complete medium**

450 mL of IMDM medium, 50 mL of 10% FCS, 5 mL of 100 U/mL penicillin/100 μg/mL streptomycin, 1.25 mL of 5 μg/mL ciprofloxacin (see Recipe 3), and 0.5 mL of 20 IU/mL IL-2 (see Recipe 4). Store at 4 °C.

**8. Complete medium with protease inhibitor**

Complete medium (see Recipe 7) supplemented with 0.5 mL of 10 µM Saquinavir. Prepare fresh and mix before use.

**9. Complete medium with protease inhibitor and DNAse**

Add 25 μL of DNase to 10 mL of complete medium (see Recipe 8). Prepare fresh and mix before use.

**10. PHA medium with protease inhibitor**

Complete medium with protease inhibitor (see Recipe 8) supplemented with 2.5 mL of 1 μg/mL PHA (see Recipe 1). Store at 4 °C. Mix before use.

**11. NaOH (10 M)**

Weigh 40 g of NaOH and dissolve in small quantities in 150 mL of MilliQ H_2_O. Add MilliQ H_2_O up to a final volume of 250 mL. Store at RT.

**Caution**: Dissolving NaOH generates heat; therefore, NaOH is dissolved in small quantities to obtain the final concentration. 10 M NaOH is a corrosive acid. Handle with care and use proper personal protection like gloves.

**12. Tris-H_3_PO_4_ pH 7.8 (1 M)**

Dissolve 21.9 g of Trizma phosphate monobasic in 60 mL of MilliQ H_2_O. Adjust pH to 7.8 using 10 M NaOH. Add up to a final volume of 100 mL with MilliQ H_2_O. Store at RT.

**13. Triton X-100 (25%)**

Dilute 25 mL of Triton X-100 with 75 mL of MilliQ H_2_O. Store at RT.

**14. DTT (1 M)**

Dissolve 154.2 mg of DTT in 0.5 mL of MilliQ H_2_O. Store at -20 °C.

**15. MgCl_2_ (1 M)**

Dissolve 9.522 g of MgCl_2_ in 80 mL of MilliQ H_2_O. Add up to a final volume of 100 mL with MilliQ H_2_O. Store at RT.

**16. Disodium pyrophosphate (Na_2_H_2_P_2_O_7_) (50 mM)**

Dissolve 1.1097 g of disodium pyrophosphate in 100 mL of MilliQ H_2_O. Store solution at RT.

**17. ATP (50 mM)**

Dissolve 275.6 mg of ATP in 10 mL of MilliQ H_2_O. Store solution as aliquots of 250 μL at -20 °C. For single use only.

**18. Pre-LAR (recipe for 50 mL)**

**Table:** 

Reagent	Final concentration	Quantity or Volume
Tris-H_3_PO_4_ (pH 7.8) (1 M) (see Recipe 12)	38.9 mM	2.25 mL
100% glycerol	0.39%	22.5 mL
25% Triton X-100 (see Recipe 13)	0.03%	6 mL
DTT (1 M) (see Recipe 14)	2.6 μM	50 μL
MilliQ H_2_O		19.2 mL
Total		50 mL

**19. Pre-LAR + D-Luciferin (recipe for 50 mL)**

**Table:** 

Reagent	Final concentration	Quantity or Volume
Pre-LAR		50 mL
D-Luciferin	0.83 mM	33.33 mg
Total		50 mL

**20. LAR substrate (per plate)**

**Table:** 

Reagent	Final concentration	Volume for one 96-well plate
Pre-LAR		1.6 mL
MgCl_2_ (1 M) (see Recipe 15)	18.7 mM	56 μL
MilliQ H_2_O		325 μL
Na_2_H_2_P_2_O_7_ (50 mM) (see Recipe 16)	0.78 µM	4.67 μL
ATP (50 mM) (see Recipe 17)	0.83 mM	50 μL
Pre-LAR + D-Luciferin		1 mL

**Laboratory supplies**

1. White 96-well plates (Thermo Scientific, catalog number: 136101)

2. 96-V bottom plates (GreinerBio, catalog number: 651101)

3. 24-well culture plates (GreinerBio, catalog number: 662160)

4. T75 vented culture flasks (Sarstedt, catalog number: 83.3911.002)

5. 50 mL polypropylene tubes (Corning, catalog number: 430829)

6. 15 mL polypropylene tubes (Corning, VWR, catalog number: 734-0451)

7. Stripettes 5 mL, 10 mL, 25 mL (Corning, catalog numbers: 4487, 4488, 4489)

8. Dual-chamber slides for TC20 automated cell counter (Bio-Rad, catalog number: 1450003). Alternative: Burker-Turk counting chambers (VWR, catalog number: BRND719505)

9. Pipette tips 20–200–1000 μL (Westburg, catalog numbers: WB 5020L, WB 5040L, WB 5074S)

10. 1.5 mL or 2 mL Eppendorf tube (sterile) (VWR, catalog numbers: 211-2130, 211-2120)

## Equipment

1. Flow cytometer (BD Biosciences, model: BD FACSCanto^TM^)

2. Luminometer (Berthold Technologies, catalog number: 1015-277R1, model: LB942 Tristar2)

3. TC20 automated cell counter (Bio-Rad, catalog number: 1450102)

4. Incubator (Thermo Fisher, model: Thermo Forma Direct Heat CO2 incubator type 321)

5. Pipettes (Gilson, p20, p200, p100; Eppendorf p10)

6. Multichannel pipette, 12 channels for 5–50 μL and 30–300 μL (Thermo Scientific catalog numbers: 4661050N and 4661070N)

7. Light microscope (Leica, model: DM IL HC Bio Microscope with table, catalog number: 11521227)

8. Water bath (Fisher Scientific Emergo, model: Schudwaterbad SW-22 catnr: 608044)

9. Pipette boy (Life Technologies Europe BV, catalog number: 9521, model: S1 Pipet Fillers)

10. Centrifuge for Eppendorf tubes (Eppendorf, model: centrifuge 5417R 230V “Refresher”)

11. Centrifuge for 15 and 50 mL tubes (Hettich, model: ROTANTA/460 RS)

## Software and datasets

1. BD FACSDiva^TM^ software (BD Biosciences) (license needed)

2. FlowJo software (Tree Star, version 10) (license needed)

3. Microsoft Excel (Microsoft Corporation, version 2016, release date: 09/22/15) (license needed)

4. GraphPad Prism (GraphPad Software Inc., version 10, release date: 07/11/23) (license needed)

## Procedure

**A. Thaw PBMCs (Day 1)**

When selecting stored PBMC samples from PWH, note that 8.2 × 10^5^ live PBMCs are required for every condition (compounds or untreated) tested.

1. Take the necessary vials of stored PBMCs from PWH from the liquid nitrogen container and immediately thaw the cells in a water bath set at 37 °C.

**Caution**: All steps must be performed inside a biosafety cabinet.

2. Transfer the PBMCs to a 50 mL tube and add 35 mL of cold thaw medium (see Recipe 5) dropwise to the cells while swirling the tube.

3. Let the cells rest for 10 min at RT.

4. Centrifuge the 50 mL tubes containing the PBMCs in thaw medium at 400× *g* for 10 min at RT.

5. Remove the supernatant by aspiration or pipetting and gently resuspend the cell pellet in 1 mL of complete medium with protease inhibitor and DNase (see Recipe 9). Add more complete medium when the cell number is expected to be higher than 20 × 10^6^ PBMCs.

6. Slightly loosen the cap of the 50 mL tubes to allow gas exchange but ensure it remains secure. Incubate the cells at 37 °C for 2 h in a humidified incubator with 5% CO_2_.

7. Centrifuge the 50 mL tubes containing the PBMCs at 400× *g* for 10 min at RT.

8. Remove the supernatant and resuspend the cell pellet in 1 mL of complete medium with protease inhibitor (see Recipe 8).

9. Count the viable cells via your preferred method.

We use trypan blue and the TC20 automated cell counter using disposable counting slides to count live cells. The TC20 has an optimal counting range between 5 × 10^4^ and 1× 10^7^ cell/mL. To stay within the optimal range, the cell suspension is diluted as follows: 10 μL of the cell suspension is pipetted into a 1.5 mL Eppendorf tube and diluted with 90 μL of PBS (1:10 final dilution). 10 μL from the diluted cell suspension is mixed with 10 μL of trypan blue in a 1.5 mL Eppendorf tube. Load 10 μL in the slide for the TC20 automated cell counter and count the live cells.

*Notes:*

*1. Cells can be counted without an automatic cell counter in the same way using a cell counting chamber or disposable cell counting slides.*

*2. The dilution can be adjusted as needed based on the cell count.*

*3. Cell viability should be >75% to obtain reliable results.*

10. For each condition (compounds or untreated), 8.2 × 10^5^ PBMCs are required for the HIVRRA. An untreated condition and a solvent-only control need to be included in every experiment.

11. Plate 8.2 ×10^5^ PBMCs for each individual condition per well in a 24-well plate. Add the compounds to the well for each of the test conditions. Adjust the volume with complete medium to 500 μL and add the protease inhibitor.

12. Culture the cells in medium alone (untreated) and in the presence of the compounds in the humidified incubator with 5% CO_2_ at 37 °C for 2 days.

*Notes:*

*1. Propagation of HIV-1 in cultures using PBMCs from PWH needs to be performed in a BSL-3 facility and requires proper training.*

*2. TZM-BL cells are required on Day 4 of the protocol. Therefore, it is advisable to passage them on Day 1 to ensure that enough cells are available on Day 4. Approximately 2 × 10^6^ cells are required per condition (compounds or untreated).*

*3. In a BSL-3 facility, liquid waste has to be inactivated before disposal. Consult the local biosafety officer, whose method should be used at your facility. Detergent or bleach treatment is usually recommended.*

**B. Compound cytotoxicity assessment and PHA stimulation (Day 3)**

Thaw PBMCs (from healthy donors) and label the cells with CellTrace^TM^ Violet (CTV) to determine the ratio between cells from healthy donors (fluorescent CTV+) and PHW (CTV-) by flow cytometry.

1. Take the necessary vials of stored healthy donor PBMCs from the liquid nitrogen container and immediately thaw the cells in a water bath set at 37 °C or use freshly isolated PBMCs. Approximately 1 × 10^6^ PBMCs from a healthy donor are needed per condition (compounds and untreated).

2. Transfer the PBMCs to a 50 mL tube and add 35 mL of cold thaw medium (see Recipe 5) dropwise to the cells while swirling the tube.

3. Let the cells rest for 10 min at RT.

4. Centrifuge the 50 mL tubes containing the PBMCs in thaw medium at 400× *g* for 10 min at RT.

5. Remove the supernatant from the cell pellet, resuspend the cells with 35 mL of RT PBS, and centrifuge at 400× *g* for 10 min at RT.

6. Remove the supernatant and resuspend the cells in 1 mL of PBS. Count the cells as described in step A9.

7. Dilute the cells with PBS to a final concentration of 10 × 10^6^ PBMC/mL.

8. Dilute 1 μL of CTV in 5 mL of PBS (or more if you have more than 50 × 10^6^ PBMCs).

9. Resuspend PBMC gently in diluted CTV: gently tap against the tube to loosen the cell pellet and add approximately 0.5 mL of diluted CTV. Resuspend the cells by up and down pipetting (5 times) using a 1 mL single-channel pipette. Dilute with diluted CTV to a final concentration of 10 × 10^6^ PBMCs/mL. Add 2.5 μL of DNase/mL to prevent the cells from clumping together.

10. Incubate at 37 °C in the incubator for 20 min, protected from light.

11. Protect the CTV-labeled cells from direct light from this point on.

12. Add 5× volume complete culture medium (RT) (see Recipe 7), mix, and incubate at RT for 5 min.

13. Centrifuge the cells at 400× *g* for 5 min and remove the supernatant.

14. Resuspend the cells in 15 mL of complete culture medium (RT) (see Recipe 7). Centrifuge the cells at 400× *g* for 5 min and remove the supernatant.

15. Resuspend the cell pellet in fresh, pre-warmed complete culture medium (1–2 mL) and incubate for another 10 min at RT before handling them again.

16. Count the cells as described in step A9. Add complete culture medium to a final concentration of 1.64 × 10^6^/mL.

**B1. Stimulate PWH PBMCs with PHA**

1. Collect the PWH PBMCs from the 24-well plate from Day 1 in sterile 1.5 or 2 mL Eppendorf tubes (one Eppendorf tube per well or condition). Add 0.82 × 10^6^ CTV-stained PBMCs per well (0.5 mL).

2. Wash the cells by centrifuging at 250× *g* for 10 min to remove any remaining medium containing the test compounds.

3. Remove the supernatant and resuspend the pellet in 1 mL of PHA medium with protease inhibitor prewarmed at 37 °C.

4. Place the cells in a 24-well plate.

5. Take 120 μL of the PWH PBMCs and CTV+ PBMCs mixture from the 24-well plate for all conditions (compounds or untreated) and transfer to 1.5 mL Eppendorf tubes (+/- 98,400 CTV+ PBMCs) to determine cytotoxicity. Place the rest of the cells in a humidified incubator with 5% CO_2_ and incubate at 37 °C for 2 days.

*Notes:*

*1. The supernatant collected in step B1.1 can be saved for other assays, such as ELISA, to determine, for instance, cytokine production.*

*2. The 24-well plate from Day 1 can be reused for Day 3 PHA stimulation if the plate is rinsed with PBS to remove any remaining medium containing the test compounds.*

**B2. Determine cytotoxicity of the compounds with flow cytometry**

**Figure 1.**
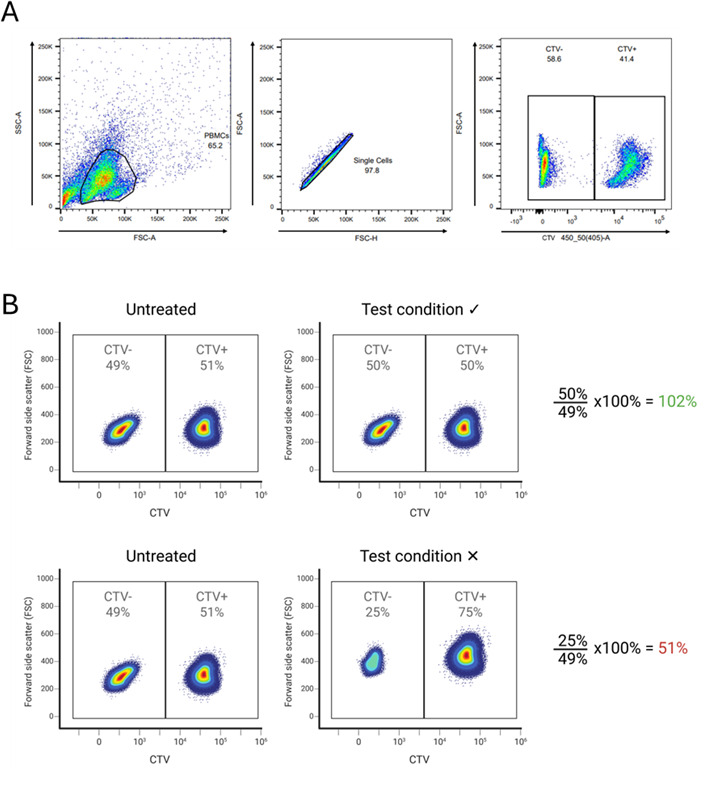
Determine cytotoxicity of compounds for peripheral blood mononuclear cells (PBMCs) from people living with HIV-1 (PWH) (CTV-) by the addition of CTV+ healthy donor cells. (A) Gating strategy CTV. (B) Top panel: In the untreated condition, where CTV- PWH PBMCs were not treated with any compounds, the proportion of CTV- (PWH) and CTV+ (healthy donor) PBMCs is similar, indicating that there was no reduction in the number of PBMCs from PWH during the assay. In the test condition, where CTV- PWH PBMCs were treated with a compound V, a similar proportion of CTV- (PWH) and CTV+ (healthy donor) PBMCs was observed. This indicates that the compound was not toxic to the PWH PBMCs that had been treated with the compound. Bottom panel: No toxicity was observed in the untreated condition, as similar proportions of CTV- (PWH) and CTV+ (healthy donor) PBMCs were observed. In the test condition, the proportion of CTV- PWH PBMCs that were treated with a compound X was decreased, indicating that the tested compound was toxic to the PWH PBMCs.

1. Continue with the 120 μL of PBMC mixture from step B1.5: this cell suspension contains 98,400 healthy donor PBMCs that have been fluorescently labeled with CTV, together with the PBMCs from PWH that have been untreated or treated with compounds or solvents only. Flow cytometry will be used to measure CTV+ (healthy donor PBMC) and CTV- (PWH PBMC) for each condition (compounds or untreated) to determine cytotoxicity of the compounds. High toxicity of a compound will lead to low concentrations of PWH PBMCs in the mixture. In untreated conditions, no toxicity is expected, leading to equal numbers of CTV+ (healthy donor) and CTV- (PWH) PBMCs (Figure 1).

2. Add 1 mL of PBS to 120 μL of the CTV- PWH PBMCs and CTV+ healthy donor PBMCs mixture (from step B1.5).

3. Centrifuge the cells at 400× *g* for 5 min, aspirate the supernatant using a pipette, and resuspend the cells in 1 mL of PBS.

4. Centrifuge the cells again at 400*× g* for 5 min, remove the supernatant, and add 50 μL of FluoroFix^TM^ buffer to fix the cells. Keep the cells at RT for at least 15 min before you determine the ratio of CTV+ (healthy donor PBMCs) and CTV- (PWH PBMCs) using flow cytometry.

*Note: FluoroFix^TM^ is a ready-to-use fixative that contains paraformaldehyde. Handle with caution and avoid direct contact.*

5. Transfer the cells into FACS tubes and measure CTV with the 405 (450/50) laser. Gate the cells according to Figure 1 to determine the %CTV- (PWH PBMCs) and %CTV+ (healthy donor PBMCs).

6. To determine cytotoxicity of a test compound, the %CTV- cells (PWH PBMCs) from the test condition should be divided by %CTV- cells (PWH PBMCs) of the untreated condition from the PWH. The percentage determines the cytotoxicity of the treatment for PWH PBMCs (Figure 1).

**C. Plate TZM-BL (Day 4)**

1. Prepare, for each plate, 10 mL of cell line medium with 2 × 10^6^ TZM-BL cells. Seed 100 μL of TZM-BL cells per well, except for the last 4 wells (column 12, E–H), which should only contain 100 μL of medium without cells to serve as background controls.

2. Incubate at 37 °C overnight in a humidified incubator with 5% CO_2_.

*Notes:*

*1. TZM-BL cells are adherent cells that need time to attach to a surface and are cultured in cell line medium (see Recipe 6). To prepare for co-culture with PBMCs, seed the TZM-BL cells onto white 96-well plates one day in advance.*

**Figure 2.**
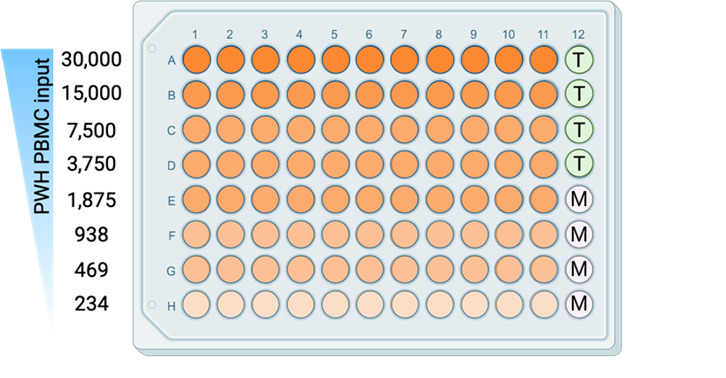
Plate layout for plating peripheral blood mononuclear cells (PBMCs) from people living with HIV-1 (PWH) together with TZM-BL cells. Each well (orange) contains CTV+ healthy donor PBMCs and CTV- PWH PBMCs, with an input of 30,000–234 only counting PWH PBMCs per well. T: TZM-BL only; M: Medium only.

*2. Per condition (compounds or untreated) of the HIVRRA, one 96-well plate will be used with 20,000 TZM-BL cells per well, except for 4 wells (column 12, E–H). See Figure 2 for a plate layout.*

**D. Co-culture PBMCs with TZM-BL (Day 5)**

1. Retrieve the 24-well plate containing the PWH PBMC and CTV+ PBMC co-culture from Day 3 from the incubator and collect each test condition in sterile 1.5 or 2 mL Eppendorf tubes.

2. Centrifuge the cells at 400*× g* for 5 min, remove the supernatant using a pipette, and resuspend the PBMCs per condition in 1.32 mL of complete medium (see Recipe 7) prewarmed at 37 °C.

3. This cell suspension will contain 720.000 CTV+ healthy donor PBMCs; the untreated condition will have a similar amount of CTV- PWH PBMCs. During data analysis, a correction for cell loss during the procedures can be performed. For this, it is essential to count the cells as described in step A9. The proportion of CTV+ healthy donor PBMCs and CTV- PWH PBMCs for each condition is determined as described in step B2.6. For the PBMC-TZM-BL co-culture, PBMC from PWH should be titrated to a cell density of 30,000–234 cells per well per test condition (see Figure 2).

*Note: The total number of PBMCs added to the TZM-BL cells will be higher because the sample contains CTV+ healthy donor PBMCs and CTV- PWH PBMCs. For the cell dilutions, the density actually starts at 60,000 cells/well.*

4. Take a 96-well V-bottom plate (one for each test condition) and add 110 μL of complete medium per well.

5. Resuspend and add 110 μL of cell suspension (60.000 CTV+ healthy donor PBMCs and 60.000 CTV- PWH PBMCs) from step D2 to row A1–A11 of the 96-well V-bottom plate. To well A12, only add 110 μL of complete medium. Row A should now have 220 μL of complete medium and cells (except for A12), and the rest of the wells should have only 110 μL of complete medium.

6. Resuspend the cells and take 110 μL from row A1–A11 using a multichannel pipette to row B1–B11. Resuspend the cells and take 110 μL from row B1–B11 to row C1–C11.

7. Repeat step D6, transferring 110 μL from row C to D and so on, until you have reached row H. Discard after mixing the leftover 110 μL from row H.

8. The cells are now serially diluted in 11 replicates across 8 rows in a 2-fold dilution (see Figure 2).

9. Take 100 μL from every well (resuspend to make sure not all cells have collected at the bottom of the plates) of the plate containing the serially diluted PBMCs and add it onto the corresponding wells of the white plates containing the TZM-BL cells (see Figure 2 for a plate layout), with the number of PBMCs (CTV+ healthy donor PBMCs and CTV- PWH PBMCs) ranging from 30,000 down to 234 cells per well.

10. Culture at 37 °C for 4 days in a humidified incubator with 5% CO_2_.

**E. Luciferase assay (Day 9)**

1. Make fresh LAR substrate (see Recipe 20) just before you perform the luciferase assay and are ready to measure luminescence.

2. Add 25 μL of LAR substrate to the wells of the TZM-BL cells using a 12-channel multichannel pipette and measure the luminescence immediately using a luminometer set at 0.5 s per measurement.

*Note: The LAR substrate is light sensitive, and therefore, luminescence should be measured immediately.*

3. Export luminescence data in Excel for data analysis.

## Data analysis

1. First, determine how many PWH PBMCs were added to the experiment. The first row should have a total cell number of 60,000, consisting of 30,000 PWH PBMCs (CTV-) and 30,000 CTV+ healthy donor PBMCs. However, test conditions might have reduced or increased the number of PWH PBMC in the culture, and this needs to be corrected with the percentage of CTV- cells. The exact cell count, determined in step C3, is expressed as cells/mL. Of this cell suspension, 110 μL was pipetted into row A, which also contains 110 μL of medium. After gently mixing, 110 μL of the cell suspension was pipetted into row B. To calculate the total number of cells (CTV+ healthy donor PBMCs + CTV- PWH PBMCs) in each well from row A, the following formula can be used:

*Formula: (PBMC count in cells/mL from C.3)/1,000 (µL) × 110(µL)/2 = input PBMCs per well in the first row (row A)*

The input of PWH PBMCs can now be calculated by the proportion of CTV- cells of the total PBMCs as determined in step B2.6.

*Formula: (Total PBMCs per well in row A) × (%CTV-/100%) = input PBMCs per well in the first row*

For example: The cell count of the cell suspension obtained in step C3 was 1.09 × 10^6^ PBMCs/mL. Each well in row A contains (1.09 × 10^6^)/1000 × 110/2 = 60,000 PBMCs (CTV+ healthy donor PBMCs + CTV- PWH PBMCs). The proportion CTV+ healthy donor PBMCs was 60%, and the proportion CTV- PWH PBMCs was 40% (step B2.6). The PWH PBMC input in each well of row A is (60,000) × (40%/100%) = 24,000 PBMCs. Thus, the second row will have 24,000/2 = 12,000 PWH PBMCs, and so on.

2. Next, for each plate, the raw relative light unit (RLU) data is converted into percentages. Begin by verifying that the medium-only rows produced no signal, ensuring there is no background signal that could have interfered with the measurements.

3. Per plate, calculate the mean of the row with the highest RLU values (the highest row-mean) and the mean from the lowest row (row H). The mean value of row H should approximate the RLU values of the wells containing only TZM-BL cells. This step ensures that the limiting dilution was effective and that the lower row does not contain enough HIV-1-infected cells to produce a signal. The mean lowest RLU signal is considered the background signal and is calculated for each plate in the experiment and used for correction.

*Note: The first (few) rows can sometimes have really low values due to cell death. Therefore, the highest RLU mean is sometimes found in row B or C.*

**Figure 3.**
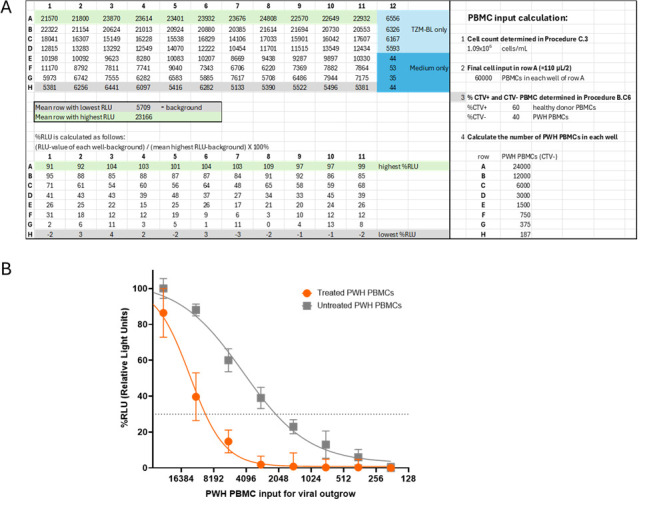
Data analysis. (A) Raw relative light unit (RLU) data are shown at the top of the Excel sheet. These values are used to determine the mean of the lowest RLU values (background) and the mean of the highest RLU values. The lower section of the Excel sheet displays the percentages calculated using the formula ((RLU value – background)/(mean max row – background)) × 100%, converting the RLU values to a scale ranging approximately from 100% to 0%. In the right panel, the calculation of the final number of peripheral blood mononuclear cells (PBMCs) from people living with HIV-1 (PWH) per well is shown. (B) Graph showing nonlinear regression analysis. Each datapoint with error bar represents mean and standard deviation for each cell dilution (11 replicates). For the calculation of the reservoir size, 30% of the maximum (dotted line) was used (online EC calculator: https://www.graphpad.com/quickcalcs/ecanything1/). In the untreated condition (gray), 1 in 2417 PBMCs contained a replication-competent virus, and thus the reservoir size is 414 IUPM. In the treated conditions, a decrease in the viral reservoir was observed (orange), and 1 in 9981 PBMCs contained a replication-competent virus, corresponding to a reservoir size of 100 IUPM.

4. Calculate the percentage of RLU for the rest of the plate based on these highest and background (lowest) values using the formula. See Figure 3A for an example.

*Formula: ((RLU value of each well – background RLU)/(mean highest RLU row – background RLU)) × 100% =%RLU*

*Note: Percentages can fall below 0%, and this is acceptable (see Figure 3A).*

5. The calculated percentages are used for logistic regression analysis to calculate the infectious units per million cells or IUPM (Figure 3B). Logistic regression analysis can be performed using, for instance, GraphPad.

6. The IUPM should be calculated from the cell dilution that contains only 1 infected cell per well. The cell dilution that gives a RLU signal of 30% of the maximum RLU has, according to the Poisson distribution, the most likely chance that the signal is caused by one infected cell per well, and thus gives the most candid reservoir prediction.

7. The number of PBMCs that give 30% of the maximum RLU can be calculated using the online EC30 calculator (https://www.graphpad.com/quickcalcs/ecanything1/).

8. The following formula can be used to determine the IUPM per condition you have:

*1 × 10^6^/cell input at 30% signal = IUPM*

## Validation of protocol

The HIVRRA uses a large number of replicates (11) to ensure precision and to measure smaller differences between the test conditions (compounds or untreated). Also, the use of an 8-fold titration enhances the reliability of the results, providing greater confidence in detecting smaller differences between the compounds (see Data analysis).

Compounds should be tested on PBMCs from a sufficient number of PWH to obtain reliable results and account for donor variation.

Untreated conditions and solvent-only controls should be included in each experiment.

Viral reservoir size is calculated using the percentage of RLU calculated based on the minimum and maximum RLU per plate rather than the raw RLU data. This limits plate-to-plate variations as each condition is measured in a separate plate, and the RLU is influenced by the time between the addition of the luciferase substrate and the luminescence measurement.

This protocol (or parts of it) has been used and validated in the following research articles:

- Jansen et al. [13]. Noncanonical-NF-κB activation and DDX3 inhibition reduces the HIV-1 reservoir by elimination of latently infected cells ex-vivo. *Microbiol Spectr* (Figures 3 and 4).

- Vlaming et al. [12]. Synergistic Activity of Second Mitochondrial-Derived Activator of Caspases Mimetic with Toll-like Receptor 8 Agonist Reverses HIV-1-Latency and Enhances Antiviral Immunity. *Int J Mol Sci.* (Figure 4).

## General notes and troubleshooting

**General notes**

1. When working with HIV-1 infected cells, all experiments must be performed in a Biosafety Level-3 laboratory (BSL-3).

2. Supernatant on Day 3 and/or Day 5 can be saved and used for ELISA.

3. The cell input can be scaled up easily, for example, to use in PCR.

4. This protocol has been validated using cells from PWH who started ART during chronic HIV-1 infection and therefore have a relatively large viral reservoir. It should be noted that the assay may not be suitable to determine the viral reservoir size using PBMC from PWH treated very early in infection, as these PWH may have a very small viral reservoir. The upper limit of PWH PBMCs in this assay is around 60,000 PBMCs per well. Adding higher numbers of PBMCs per well will increase cell death of the TZM-BL and will reduce reliability.

5. Freshly isolated PBMCs from PWH (and healthy donors) can also be used. Contamination of isolated PBMCs with high concentrations of thrombocytes or erythrocytes may affect the assay. It is recommended to include additional washing steps after isolation of PBMCs to remove thrombocytes and lyse red blood cells, using, for instance, red blood cell lysis solution from Miltenyi Biotec (catalog number: 130-094-183).

6. Instead of using the in-house LAR substrate, you can also use commercially available substrates for luciferase reporter assays, like the luciferase assays from Promega (catalog number: E1501) or QUANTI-Luc^TM^ from InvivoGen (catalog number: rep-qlc4lg1).

**Troubleshooting**

Problem: Nonlinear regression lacks a smooth S-curve, making calculations more difficult.

Possible causes:

1. Cell death in the first (few) rows results in fewer data points.

2. Titration was not done properly.

3. The number of cells harboring an inducible replication-competent virus in PWH PBMCs is high, and therefore, infected cells are detected even at the highest dilution.

Solutions:

1. For the nonlinear regression analysis, you should start with the row with the highest cell input having the highest RLU values (the highest row-mean).

2. Resuspend between serial dilutions.

3. Include additional dilutions when using PWH that may have a high number of cells containing an inducible replication-competent virus.
